# *Botlhoko, botlhoko!* How people talk about their musculoskeletal complaints in rural Botswana: a focused ethnography

**DOI:** 10.3402/gha.v8.29010

**Published:** 2015-12-17

**Authors:** Maria Hondras, Corrie Myburgh, Jan Hartvigsen, Helle Johannessen

**Affiliations:** 1 Department of Sports Science and Clinical Biomechanics, Faculty of Health Sciences, University of Southern Denmark, Odense, Denmark; 2 Nordic Institute of Chiropractic and Clinical Biomechanics, Odense, Denmark; 3 Department of Public Health, Faculty of Health Sciences, University of Southern Denmark, Odense, Denmark

**Keywords:** Botswana, communication, ethnography, interpreter, joints, musculoskeletal system, pain, qualitative research, signs and symptoms, translating

## Abstract

**Background:**

Conflicting interpretations about the structure and function of the body contribute to discordance in communication between healthcare professionals and lay people. Understanding musculoskeletal (MSK) complaints presents additional complexities when discussed in more than one language or in cross-cultural settings. In low- and middle-income countries (LMICs), few healthcare professionals have specialist MSK training and not all practitioners speak the primary language of patients.

**Objective:**

Our goal was to understand how people in rural Botswana perceive and express MSK complaints.

**Design:**

Ethnographic fieldwork for 8 months in the Botswana Central District included participant observations and interviews with 34 community members with MSK complaints. Audio-recorded interviews were typically conducted in Setswana with an interpreter, transcribed verbatim, and contextually translated into English. Abductive qualitative analysis was used as the interpretive methodology.

**Results:**

Whereas initial responses about MSK troubles yielded the exclamation *botlhoko, botlhoko!* combined with animated non-verbal gestures and facial expressions indicating widespread body pains, in-depth interviews revealed the complexities of pain expression among respondents. MSK pains were described as ‘bursting, exploding, aching, numbness, hot, pricking, stabbing, swollen, and pain in the heart’. Language subtleties manifested during interviews, where ‘meat’ or ‘flesh’ implied soft tissue pains; waist pains were voiced yet portrayed as low back or sacroiliac pain; and ‘veins’ variously referred to structural and functional types of pain. Psychological and social stressors accompanied many accounts of MSK troubles.

**Conclusions:**

Respondents offered diverse MSK symptom descriptions consistent with biopsychosocial illness models, yet few communicated complaints using the biomedical language of healthcare providers. Although research interview and transcription processes may not be practical for clinicians, working with interpreters who communicate detailed patient accounts for MSK troubles will complement patient–provider encounters. Community member perceptions of their MSK pain and associated conditions should be explored and incorporated into healthcare interventions and innovations for rural communities in LMICs.

For the millions of people worldwide who suffer from musculoskeletal (MSK) conditions, the quality of communication about their pain (1) and its impact on their everyday life affect the health trajectory of their illness (2). Researchers argue for more patient-centred strategies (3–6) and outcomes (7–10) to assess and treat people with MSK conditions. Although the biopsychosocial concept of illness was proposed as an operational clinical model for low back pain and disability more than 25 years ago (11), researchers continue to voice the criticism that ‘not all components of the trinity are well-represented’ (12), not only for outcome measures, but also in clinical practice where the biomedical model predominates during healthcare communications and decision-making (13–15).

Cedraschi and colleagues called for a shared language between back pain patients and providers that ‘requires a shift from medical and scientific classifications and terminology to a language comprehensible to lay people’ (16). This shift in communication skills requires the provider to incorporate patient perceptions, beliefs, expectations, and experiences with specific biomedical knowledge and explanations to manage patients with painful conditions. Poor communication between patients and healthcare providers has been reported during encounters with back pain patients, especially ‘the longer the patient has suffered pain’ (17).

Not only do patients and providers in high-income countries lack a common language for MSK symptoms, we know very little about how people talk about MSK pain in low- and middle-income countries (LMICs). Although research conducted in Africa advocates the importance of incorporating social, cultural, and behavioural semantics into diagnostic processes and public health education and control strategies for acute respiratory infections (18), HIV and AIDS (19), malaria (20), and tuberculosis (TB) (21, 22), few studies have evaluated languaging for MSK conditions.

In South Africa, Shaikh and colleagues (23) examined terminology used in English and isiZulu for neuropathic pain experienced by patients with a confirmed HIV diagnosis. Although there was a high level of concordance between some spontaneously expressed terms in isiZulu and terms selected from an English word list, there also was high variability in knowledge and understanding of English neuropathic terminology. In Zimbabwe, Madzimbamuto (24) used the chiShona language to name the vertebral skeleton, arguing for collaboration between scientists and linguists to develop medical terminology schema such that ‘knowledge already embedded in the (local) language is not lost’.

In Botswana, English is the official language of the government, business, health, and education sectors, yet almost 80% of the population speaks Setswana as their primary language and less than 3% speak English at home (25). To our knowledge, there are no studies examining how people express or report MSK conditions in Setswana. Qualitative studies are well suited to uncovering cultural and social contexts that give meaning to the experiences of MSK pain and disability (26) and thereby inform effective care and support for individuals and communities (27). Informed communications about MSK health and disorders can add dimension to measuring what is important to sufferers (12) and can improve the viability of healthcare delivery.

The purpose of this focused ethnography is to examine the burden of living with and caring for people who experience MSK conditions among rural *Batswana* (the people of Botswana). One project aim is to understand how *Batswana* perceive and talk about MSK pain. Clear understanding of *Batswana* perceptions for MSK pain has practical implications for healthcare providers and lay caregivers, as well for communities developing and engaging in healthcare strategies for rural community members. In this paper we examine expressions for MSK symptoms among persons living in two rural communities in Botswana's Central District.

## Setting and methods

In 2012, a non-governmental organisation (NGO) established two spine care clinics in Botswana's Central District aiming to develop a low-cost model of care for primary spine care clinicians, educate local healthcare workers and patients, and conduct research (28). Two of the authors (MH and JH) joined the NGO research team to provide empirical data about MSK burden in the local context.

Ethnographic material was generated during three visits to the Central District: 1 month in late 2011, 1 month in early 2013, and 6 months from October 2013 to March 2014. The community context and methods for this qualitative study are described elsewhere (29).

### Participant recruitment and data collection

Respecting the mode of communication of the *dikgosi* (‘village chiefs’), we introduced the project at scheduled *kgotla* (‘community meeting place’) gatherings. Purposive sampling was used to invite participants regardless of age, gender, social status, or physical condition, where we sought information-rich cases to illuminate the research aims under study (30). Participants were rural community members who suffered from or cared for people suffering from MSK conditions, including people who had or had not attended the NGO spine care clinic.

Non-participant and participant observations and informal interviews, both with and without study interpreters, were conducted in settings where people engaged in daily routines. In this way, the researcher interacted and participated with people during work and leisure time to observe and talk with them about what they were doing, thinking, and saying (31) in relation to MSK health.

In-depth interviews averaged 60 minutes and were conducted in people's homes, outdoor living areas, open and closed spaces at the *kgotlas*, clinic facilities, and health posts. Digital audio recordings were transcribed verbatim in the primary language spoken and then contextually translated into English. Because equivalent meanings are not always possible with verbatim translations from Setswana to English, transcriptionists aimed for contextual consistency rather than verbal consistency (32). MH augmented transcripts with notations of non-verbal behaviour (33) generated as field notes during interviews.

### Data analysis and interpretation

We used abductive reasoning for data analysis and interpretation (34). Abduction in qualitative analysis is a combination of inductive and deductive thinking with logical underpinnings [(35) citing (36)]. More recent applications distinguish abduction from data-based inductive analysis and theory-derived deductive analysis demonstrating the continuous reflexive and dialectic exchange between theories, analytical concepts, and empirical findings (37–39).

During fieldwork and throughout data analysis, MH consulted with respondents and key informants who spoke Setswana as their first language (including interpreters, transcriptionists, *dikgosi*, health education assistants, and nurses) to examine her evolving understanding of the cultural context and meaning of her observations and interviews. How respondents languaged their MSK symptoms was a point of repeated clarification during these discussions.

Upon returning from the field, NVIVO data analysis software was used to manage the iterative phases of data analysis. Initial stages included close reading of interview transcripts, field notes, and memoranda by the first author to generate substantive concepts that made sense of the data for how respondents described and explained their MSK troubles, broadly clustered into the domains *MSK perceptions* and *Glossary of MSK terms*. Secondary, tertiary, and subsequent readings used constant comparison (40) to identify similarities and differences in these domains within and across cases, with frequent returns to the raw data to review concepts and reflect on patterns and connections with the data in an inductive manner. Subsequent review of the literature incorporated theoretical perspectives about verbal and non-verbal expressions of pain in a deductive manner, allowing further refinement about how *Batswana* express MSK troubles.

Although technological challenges in rural Botswana prohibited communication with international collaborators during fieldwork, MH regularly reviewed and discussed analysis and interpretation with co-authors, key informants, and professional colleagues. All authors reviewed the conceptual framework for this project aim and agreed to synthesise these data into three analytic categories: the language of MSK pains; non-verbal expressions of pain; and biopsychosocial talk for MSK troubles.

### Ethical considerations

The Health Research and Development Committee (Botswana Ministry of Health) granted ethics approval (HRDC 00735) for this study. The *dikgosi* from the three main wards also granted permission to conduct the study in their village. We obtained written informed consent and permission for audio recordings before the first interview with each participant. Women are addressed as *Mma* and men as *Rra* in Botswana; this convention along with sequential letters of the English alphabet is used for participant pseudonyms.

## Findings

Findings are based on 55 interviews with 34 adult participants (25 women, 9 men) whose ages ranged from 20 to 97 years. During the pretest phase participants talked about MSK ‘troubles’ or ‘problems’ to describe symptoms as opposed to ‘pain’. These terms are used interchangeably in the findings.

### The language of MSK pains – botlhoko and more

Participant accounts created challenges for understanding the tissue type, location, and nature of symptoms. When asked ‘tell me about your *mesifa*, *marapo le ditokololo*’ (muscles, bones, and joints), the majority of participants exclaimed, *botlhoko, botlhoko!*, implicating whole-body pains with their initial responses and gestures.

The Setswana word *botlhoko* is commonly used for pain, yet has distinct meanings in English [‘disease; illness; pain; ache; throb; bitterness; spiritual anguish’ (41, 42)] encompassing biological, social, sensory, and spiritual experiences that may not be dissociated in the mind of the sufferer. Thus, the expression *botlhoko* required further questioning about attributes and contextual relationships.

Participants ultimately expounded on the nature of *botlhoko*, using words such as *thunya* (‘on fire, exploding, bursting’), *phaphanya* (‘aching’), *bolelo* (‘hot’), *bosisi* or *bogatsu* (‘pins and needles; numbness; cramp; tingling’), *tsunyetsa* (‘pricking’), *setlhabi* (‘stabbing’), and *rurugile* (‘swollen’). One woman at the health post used *thunya* several times to portray pains in multiple body regions:

My body is feeling painful all over. My legs are exploding. My shoulders are exploding and this part here [bilateral flank] as well is exploding. (Mma A, age 58)

Language subtleties for anatomical references also manifested during interviews, particularly for soft tissue structures. ‘Meat’ or ‘flesh’ (*nama*) and ‘muscles’ or ‘sinews’ (PL. *mesifa* or *mesiha*; SING. *lesifa* or *lesiha*) were mentioned, almost interchangeably.

This one seems to be a muscle [*lesifa*] … it doesn't feel like a bone [*lerapo*]. Yes madam, because when I hold [places right hand on right sacroiliac region and rubs right buttocks] like this … just feel some meat [*nama*] … Yes, right bottom [buttock]. (Mma P, age 68)

One participant revealed a conspicuous example for the differing perception of pain, at least between *Batswana* and Europeans/North Americans. Mma S stood up during the interview and exclaimed:

They said these things [cupping both buttocks] are the muscles, they didn't work, the way I understand. Ai! Have you ever seen a buttock getting sick? Some of us have never heard of flesh having pain! (Mma S, age 54)

While the term ‘veins’ has specific biomedical connotations related to the cardiovascular system for health professionals, during many interviews it was not always clear whether this term referred to muscles, veins, other soft tissue structures, or radiating pain. Specifically, a common expression participants used to describe the location of their MSK pain was *tshika* (PL. *ditshika*), defined as ‘a generation; lineage; blood vessel, vein; artery’ (42).

Each and every day. There is no free day. Already it has … maybe it has spread through all my veins this thing. This pain! The pains that are on me! (Mma A, age 58)

During follow-up interviews, we asked participants to clarify their use of the word ‘veins’. Invariably respondents struggled to explain *ditshika*. Some inferred they felt tension, stiffness, or a bit of numbness, but could not identify a direct spot or area for their pain. Other respondents described ‘an electric shock’ or ‘this pain that travels’. In contrast, one woman implicated blood vessels as the source of her pain:

What gives me problems, I'm troubled by this [placing right hand on low back area]; next [places hands on left thigh, then calf]; can you see it? This is where the pain [is]. Now I don't know if this vein here is the one which usually … These veins! Can you see they are green? Yes they cause pain. (Mma L, age 47)

A striking discovery was that participants rarely referred to ‘low back pain’ or even ‘back pain’. Rather, they used the Setswana terms *dinokeng*, *dinoka*, *noka*, or at other times *letheka* – all of which literally translate into ‘waist pains’. And yet, these waist pains were depicted by placing their hands across the low back, sometimes covering their sacroiliac joints bilaterally. One man linked his waist and leg pains with blood vessels, rather than with his MSK or nervous systems as a clinician might:

Yes, the other thing that troubles me a lot is the waist; this waist [places hands bilaterally across low back] … it has become so heavy. If I just [continue working] with it, it becomes extremely heavy. And also the legs; even when the [right] knee pain decreases, I can't manage to even run. They can be so heavy that at times I fail to run; both legs can be heavy. It's like the blood vessels have hardened. Yes Madam, it's like they have hardened. (Rra G, age 65)

While waist pain and veins were expressed in the next account, with a clinician's ear, this excerpt sounded like low back and sacroiliac pain that radiates to the lower extremities:

I have problems on my waist. Usually it's on this bone [places hand over right sacroiliac joint]. The pain penetrates so much … it was all painful on my waist right here [hands across low back region]. Which means if I have a stabbing pain like that I can't stand up. Then there are the muscles over there [slides right hand across right hip region]. They get to feel like they are being pulled. And the pain travels in the veins through the leg [rubbing back of right thigh]. (Rra C, age 56)

Beyond *botlhoko*, the adjectives respondents used to describe MSK pain were vividly demonstrated during several hours of participant observation when we ‘slayed a beast’ – an ox – for consumption among four families. Respondent accounts of the meat, flesh, and veins combined with the descriptors ‘bursting’, ‘exploding’, ‘swelling up’, ‘spreads through’, and ‘stabbing and breaking apart’ were precise, graphic depictions of the intense process to slaughter the ox.

### Non-verbal expressions of pain – the body's language of botlhoko

As depicted in previous excerpts, few accounts were devoid of non-verbal expressions for MSK pain. Most respondents used animated gestures and facial expressions and placed their hands on body parts to provide another path for illuminating their embodied experience of MSK pain. Accompanying initial responses – *botlhoko, botlhoko!*, whole-body pain gestures included some variation of folding the arms across the chest, placing the hands on opposite shoulders, and rubbing the hands down the arms, then spreading their hands to the ipsilateral sides of their torso and continuing to rub down both sides of the pelvis and lower extremities.

Mma O is a 72-year-old woman who described pain that was moving in her body and used animated facial and bodily gestures to characterise her symptoms. She complained of diffuse lower abdominal pains and bloating while gesturing with both hands rubbing and sliding from her abdomen to her low back and sacroiliac regions:

Mma O: Now, this pain is only felt here [both hands lay on abdomen; facial grimace] … The pain moves from here to that side [the low back, bilaterally]. I feel it at the back. Again this body, or should I say the meat on this side of my (low) back, it's like something is moving in my body but when I look I don't see anything … and when it becomes painful I continue to press [facial grimace], I keep on pressing it [low back].

Interviewer (IV): So you have pain in your back?

Mma O: No, not the back but the meat on the back.

IV: Okay, the meat?

Mma O: Yes, yes. Now in the body I sometimes develop a small rush … producing a pricking pain [scrunches face] like that of a needle [jabbing epigastric area with her right thumb].

In this instance, as with the majority of cases, whether or not Mma O was experiencing referred pain to or from her abdomen and to or from her low back region, or clinically distinct pain and dysfunction in multiple sites, will require further in-depth communication between practitioner, interpreter, and patient to discern the relevant diagnosis(es), treatment plan, and prognosis.

### Biopsychosocial talk for MSK troubles

Verbal and non-verbal expressions for MSK conditions spoke volumes and were, in many cases, amplified with references to psychosocial elements underlying troubles in conjunction with the more biologic expressions for MSK pain. Respondents folded explanations of MSK pain into descriptions of other co-morbid symptoms or conditions, which typically were the focus of their healthcare providers’ routine clinical queries and ministrations. ‘High blood’ [pressure], diabetes, and ‘the [HIV/AIDS] virus’ were common reasons for seeking medical care, as were visual disturbances (often cataracts), gastrointestinal problems, and in a few cases TB. For example, Mma S went to the village clinic when her back and leg pain were severe. The nurses took her blood pressure and prescribed a diuretic. Mma S was at a loss about how to control her back and leg pain, as the clinic nurse did not evaluate or treat her for her presenting MSK complaints. She acknowledged that her MSK pains, blood pressure, and emotions were contributing factors to her current state of ill health. She told us:

Yes! You see? I told them, hey! This [back and leg pain] is painful, that is why my BP is up … also because I am feeling pain in my heart! I know. No! What I am saying … in my life … when I have a problem, I want to share. Yes, so, I know, you see, when the problem comes, I should share so that I don't keep it for myself.

You see with emotions, if you are in pain, that is if the pain is too much … you also become emotional. You change. I mean taking you as an example if you have too much pain, severe pain. With me I become so aggressive and the children just irritate when you tell them it's painful … I get annoyed, it makes me moody. That is why I'm saying … I noticed that sometimes if you hear a person is sick and then you're told the sickness has gone to his head, you will be experiencing this condition. It's like … you feel like beating them up. (Mma S, age 54)

In another account, Rra H talked about his headaches that used to be intermittent, often months apart, but now were occurring more frequently and with greater intensity. Here again, the healthcare provider offered a biological remedy but did not attend to how the patient felt psychosocial stressors impact his headaches. He remarked:

Like for the past two weeks I went to clinic, and they gave me painkillers. But sometimes, I might have this headache. I just think it's stress, or life stresses, and so I will take the painkillers, but they do not really help because still, the life stresses … or sometimes somebody doesn't talk to you in a proper manner, but they don't see anything wrong with it, that may cause a lot of stress, or if you have a project you want to do, and then you don't have enough money, to push the project … my head just aches. (Rra H, age 47)

Mma U is an 82-year-old woman who, according to her last born of 13 children, was ‘so hard working; she could plough and do anything’. Mma U did not know what caused her MSK pains that ‘move in the neck, the waist, and the knees’ but assumed they were caused by diabetes because, ‘after being told I was diabetic, that's when the (MSK) pains started’. Her daughter confirmed Mma U was diagnosed with diabetes 2 years prior and that was about the same time she ‘took to sitting all day’ under the shade tree in their compound and no longer worked. However, after hearing her mother attribute her MSK pains and lack of activity to diabetes, her daughter chimed in:

Why can't she say that it's because during that period we experienced a number of funerals, you see? Yes, even here at home, our children and her children passed away so we think that maybe her heart was so much affected by the problems which troubled her … so maybe the stress caused the pains. (Daughter of Mma U)

The foregoing examples provide a glimpse into how rural *Batswana* and their families think and talk about MSK pain in the context of their everyday lives. Giving people the opportunity to talk about their MSK troubles provided another path to understanding the complex relationships for the biopsychosocial model of illness.

## Discussion

Communication about MSK disorders is not only challenging when health professionals use different terminology to communicate with each other, but is also a challenge when patients and providers lack a shared understanding for communicating information (1, 16). Patient–provider encounters in cross-cultural settings increase the level of complexity related to healthcare communication. To our knowledge, this is the first report on the everyday language of MSK complaints among rural community members in Botswana, the majority of whom speak Setswana. Our findings, while rooted in a specific cultural context, are likely transferrable to rural settings in other LMICs. Through in-depth interviews, we uncovered pain semantics that may be important for healthcare practitioners, particularly in countries where there are few MSK specialists and where NGO providers who now deliver MSK care do not speak the local language(s) yet value patient education as part of their intervention strategies (28).

Whereas the majority of respondents initially expressed whole-body pains, exclaiming *botlhoko, botlhoko!* combined with animated non-verbal gestures, persistent questioning allowed people to explicate their *botlhoko* using Setswana terms. Language subtleties manifested for anatomical references, where ‘meat’ or ‘flesh’ was implicated as muscle or other soft tissue structures; ‘waist pains’ were expressed yet portrayed as low back and/or sacroiliac pain; and the term ‘veins’ was variously used to represent either anatomical structures or the nature of pain that moves or flows through body regions. In some accounts, all three components of the biopsychosocial model manifested as important dimensions for languaging MSK troubles.

Although there were diverse symptom descriptions, few respondents communicated MSK pain in the context of clinical paradigms and practices. This is not surprising given the dearth of MSK practitioners in the country. Indeed, the majority of participants in our study were not seeking care for specific MSK complaints; rather, these were co-morbid complaints communicated when seeking care for hypertension, diabetes, HIV and AIDS, or other debilitating conditions. Now that an NGO spine care clinic is in place in the villages where we conducted research, it will be interesting to see if MSK language shifts, over time, with community members incorporating more Western paradigms or whether practitioners adopt local vernacular for clinical encounters and educational strategies.

As with any clinical encounter, clinicians must translate the patient's subjective symptom expression into a pattern they recognise objectively. Often, particularly with vague symptom presentations, this translation requires negotiating discrepancies between the language(s) used by patients and the clinical terminology that practitioners bring to the clinical encounter (43, 44). In our study, while interpreters served an important role to synthesise dialogue during interviews, transcripts provided depth and breadth for clinically relevant information. From an example cited previously, transcripts revealed that Mma A said her legs, shoulders, and bilateral flank were exploding; yet, during the interview the interpreter communicated to the researcher ‘what she is saying is that she has general body pains in the shoulders and the legs are aching’.

Collaborative care that emphasises patient-centred communication and a respect for individuals as experts in their own body (45) will require extra attention by practitioners who are not fluent in the local language(s) when they work with interpreters to evaluate patients (44). Health communication strategies will be important to avoid encounters where information that patients perceive as relevant are deemed irrelevant by the provider or interpreter (46). This also points to a fertile area of research related to developing outcome measures to track patients’ progress during courses of care. ‘Simple’ forward and backward translations of previously validated or newly developed instruments will require thoughtful attention to the local context and the way people in LMICs with few MSK specialists think about and articulate their MSK troubles.

Whereas the term ‘veins’ was used in several contexts, yielding nuanced interpretations, the findings in our study are consistent with Livingston's presentation that *ditshika* may be defined as veins, channels, or nerves (47). Although none of the transcriptionists in our study specified ‘channels’ or ‘nerves’ when transcribing *ditshika*, it is important to pursue the use and understanding *Batswana* have related to veins as structures and pathways. For example, similar to Nyamanga's study (48) of the Luo in western Kenya, livestock are important components of the socio-economic and cultural lives of *Batswana*. Nyamanga and colleagues reported similarities and differences in the perceptions and practices for human and animal health among Luo farmers. In their study, illnesses were perceived as a result of an ‘inhibition of flow through blockage in the various channels that supply life to the body’ (48). In our study of rural *Batswana*, MSK talk about veins may be related to what they see and know about slaughtering livestock. Regardless of the interpretation, mapping community members’ understanding of functional and anatomical references can inform clinical encounters for MSK disorders. Practitioners must be vigilant in conversation with people about their MSK pain, not only in the context of other pathophysiologic indicators but also related to cultural concepts that will resonate with *Batswana* to move toward MSK health.

Our interviews with rural community members support the notion that MSK pain is a complex, multidimensional process that warrants embracing the biopsychosocial model of care and also incorporating the spiritual experiences and beliefs *Batswana* hold. The English meanings for *botlhoko* encompass biological, social, sensory, and spiritual experiences that may not be dissociated in the mind of the *Motswana* (a single Tswana person) sufferer. Several respondents conveyed ‘pain in their heart’, which according to Ingstad (49) is a way *Batswana* express that the person is worried or depressed and is linked to an ancestral power (*dikgaba*) that ‘may descend upon the persons (most often their children) who are the objects of worry’. As we move forward to develop clinical outcomes assessments at the NGO spine care clinic and interventions in Botswana villages to improve MSK health, we must be mindful of attending to the physical, emotional, social, and spiritual dimensions of MSK distress and, ultimately, healing relationships (50, 51). Next steps include engaging community members to design culture-centred communication (52, 53) strategies that create shared meaning (46, 54) about MSK health and conditions. We have much to learn from *Batswana* about the interconnectedness of these dimensions and how we can ‘frame what really matters’ (46) to *Batswana* as we build sustainable models and paths of care for individual patients and communities at large.

### Strengths and limitations

The major strength of this study is that we provide empirical data demonstrating that few Setswana words are used to describe MSK pain, yet these few words have varied and in-depth meanings when contextually translated into English. In addition, terms that hold anatomical and functional relevance for biomedical practitioners may not resonate with people where the biomedical vernacular for MSK conditions is uncommon. Examples from Western cultures, where patients and providers speak the same language, demonstrate that people with MSK pain report that their providers do not understand them (55), give them credit for knowing their own body (56), or necessarily use comprehensible terminology even when providers think they do (1). The clinical implications of this work for both developed and developing countries are valuable for providers and patients to not only more accurately diagnose potential medical problems but also to cultivate therapeutic partnerships.

Another strength of this study is that we designed explicit strategies to conduct the work in two languages and employed local *Batswana* as translators for written language, interpreters for spoken language, and transcriptionists to transcribe audio-recorded Setswana and provide contextual English translations. Although these methods are detailed elsewhere (29), interpretations made from transcribed and translated interviews are not without limitations. One colleague explained the difficulties in this way. ‘Even when I did my PhD [in the US], I came back to Botswana to collect the data with focus groups … even though I am fluent in Setswana and English … how do I say this in English … I will lose meaning … because the concept does not match … it happens … even for Tswana speakers. But with your methods of transcribing and translating, you will take care of some of these limitations’ (personal communication, NS, 02 April 2013).

Although we did not ask participants to review transcripts, follow-up interviews allowed us to pursue interpretations about preliminary findings with respondents. We remain cautious, however, about over-interpreting similarities and differences across cultures, given that none of the authors are fluent in Setswana. As Owusu (57) argues, the ‘one critical factor that greatly contributes to systematic errors in ethnographic accounts is the lack of language familiarity or fluency’. Nevertheless, given the lack of MSK specialists in Botswana, the high number of expatriate physicians in the healthcare sector, and the cadre of NGO volunteers who serve short terms of service without learning Setswana, we believe we uncovered language representations for MSK disorders that can influence how healthcare practitioners interact with *Batswana* in everyday practice.

## Conclusions

With this paper we give voice to the people in two rural Botswana villages for their MSK symptoms. This voice, respondents claim, has previously gone unheard and unheeded, as little information is passed about the nature, treatment, or prognosis for those who suffer with MSK pain and disability. Clear understanding of local perceptions for MSK conditions and associated symptoms has practical implications for healthcare providers and lay caregivers, as well for LMIC communities developing and engaging in healthcare strategies where few MSK specialists exist.
